# Multimodal temporal-clinical note network for mortality prediction

**DOI:** 10.1186/s13326-021-00235-3

**Published:** 2021-02-15

**Authors:** Haiyang Yang, Li Kuang, FengQiang Xia

**Affiliations:** 1 School of Computer Science and Engineering, Central South University, Changsha, 410083 China; 2 Changsha Hospital of Hunan Normal University, Changsha, China

**Keywords:** Electronic medical records, Mortality prediction, Deep learning, Multimodal learning

## Abstract

**Background:**

Mortality prediction is an important task to achieve smart healthcare, especially for the management of intensive care unit. It can provide a reference for doctors to quickly predict the course of disease and customize early intervention programs for the patients in need. With the development of the electronic medical records, deep learning methods are introduced to deal with the prediction task. In the electronic medical records, clinical notes always contain rich and diverse medical information, including the clinical histories and reports during admission. Mortality prediction methods mostly rely on the temporal events such as medical examinations and ignore the related reports and history information in the clinical notes. We hope that we can utilize both temporal events and clinical notes information to get better mortality prediction results.

**Results:**

We propose a multimodal temporal-clinical note network to model both temporal and clinical notes. Specifically, the clinical text are further processed for differentiating the chronic illness patients in the historical information of clinical notes from non-chronic illness patients. In order to further mine the information related to the mortality in the text, we learn the time series embedding with Long Short Term Memory networks and the clinical notes embedding with a label aware convolutional neural network. We also propose a scoring function to measure the importance of clinical note sections. Our approach achieved a better AUCPR and AUCROC than competing methods and visual explanations for word importance showed the interpretability improvement of the model.

**Conclusions:**

We have tested our methodology on the MIMIC-III dataset. Contributions of different clinical note sections were uncovered by visualization methods. Our work demonstrates that the introduction of the medical history related information can improve the performance of the mortality prediction. Using label aware convolutional neural networks can further improve the results.

## Background

**Fig. 1:**
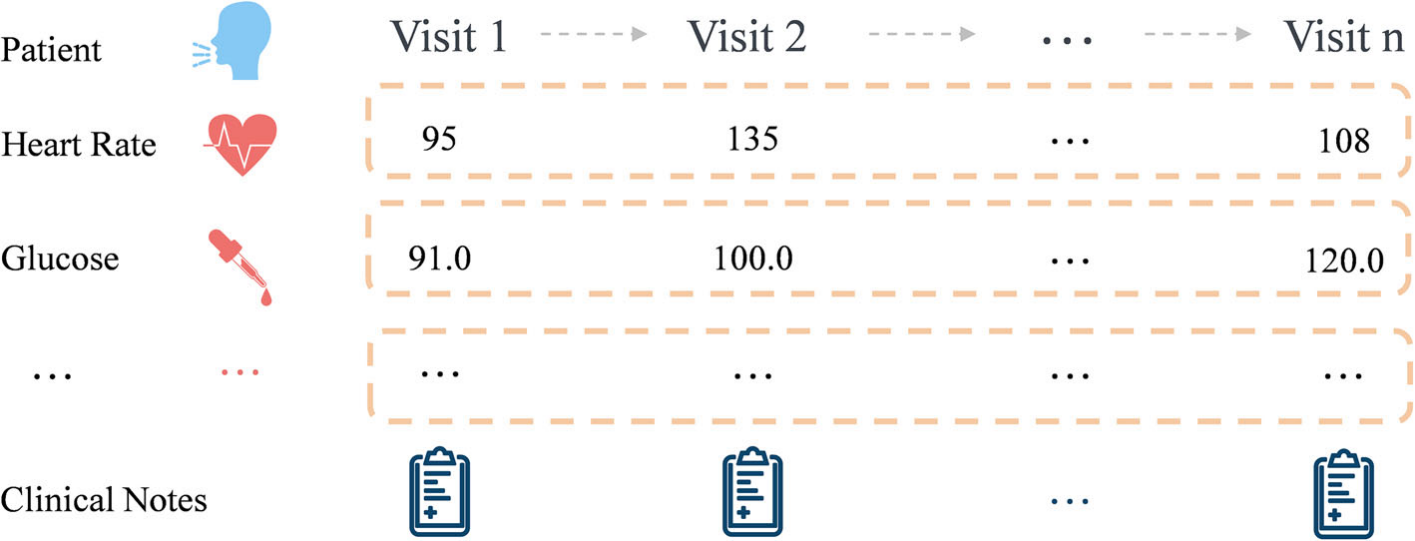
Patient visit timeline. A sample of patient electronic medical records

The development of the information science and technology makes lasting contributions to the evolution of the management of intensive care unit (ICU). With the increasing number and complexity of biosensors used in ICU, a great deal of data needs to be processed. Electronic Medical Records (EMR) consists of multitype data that records the patients’ visits in hospitals (as shown in Fig. 1). EMR contains the numerical results of physical examination in time series, which will be called time series data in the following paper. In addition to these laboratory examination data, doctors will also record patients’ relevant information as clinical notes during ward round, such as the history of present illness, social history, family history, chief complaint, clinical history, past medical history, and so on.

The field of estimation on the health status of ICU patients have produced throughout the years. Medical researchers have proposed a lot of scoring systems to evaluate the prognosis, severity and effectiveness of clinical treatments. Almost all of these systems widely applied in the hospitals refer to the specific symptoms and vital signs of patients for statistical calculation. Knaus et al. [1] established the world’s first health scoring system, as early as 1981, namely acute physiology and chronic health evaluation (Apache). APACHE II [2] is still, after many years, the most widely used and mature system, which is used to predict the mortality of ICU patients, detect and treat abnormal changes in acute physiology. However, such scoring methods mechanically divide the numerical outcomes into several established thresholds for scoring, which leads to the overestimation of mortality [3].

On the other hand, machine learning methods have demonstrated state of the art performance in modeling the mortality problem mining the time series vital data of the EMR data [4, 5]. The researchers often choose the corresponding signals of the existing medical evaluation methods mentioned above as the features to train the time series model. However, there are still a lot of multitype data in ICU database that have not been utilized effectively, especially clinical notes. In fact, in the actual medical diagnosis process, the contents involved in clinical notes are also important information that doctors need to measure. Furthermore, the history of the illness and other historical information should be considered for patients with chronic diseases, but not for people with non-chronic diseases. Specially the two research challenges are summarized as follows:

- **Making more use of the historical clinical notes:** Most of the existing studies have not introduced clinical notes into the prediction model. The model [6] considering clinical notes only took account of the contents which are synchronized with vital information. However, this part of content is partially duplicated with the information contained in time series and does not contain the patient’s historical information. In practice, the patients’ chief complaint, disease history, family history and allergy history are the important factors when doctors diagnosing. At the same time, the history of illness and other related information are treated differently for chronic patients and non-chronic ones.
- **Exploring the contribution of notes in different sections to mortality:** As mentioned above, there are many sections in clinical notes. There is no difference in the contribution degree of these sections in the model of previous work. Thus, it is crucial to learn the text representation that captures the dependencies to mortality prediction task.

To address the questions above, we propose a multi-modal deep neural network that considers the time series data and more clinical notes at the same time. Moreover, we treat chronic and non-chronic patients differently when dealing with clinical notes. The visualization results show the importance that the model assigns to each text. Our main contributions are summarized as follows:

- We first distinguish the treatment of clinical notes for chronic patients and non-chronic patients. In addition to the report during admission, the history of present illness, family history and other history information in clinical notes are introduced to the model. For the chronic patients, the medical related histories in clinical notes are taken into account. For the non-chronic ones, only the recent notes are considered. In this way, more information of clinical notes are used and more clinical text representation of patients can be extracted. We propose a label-aware CNN model to extract the text feature, in which the label attention layer can learn the clinical notes representation from the joint space. In results, the relevant words are weighted higher in the mortality prediction task than others. We visualize the weights of the words to detect the most contributing notes for the patients for providing interpretability.
- To further capture the dependencies of the clinical text in different sections, we propose a scoring function to capture the clinical note section contributions with respect to model predictions. In this way, we can understand which part of the notes has the most impact on mortality.
- We evaluate the effectiveness of the proposed model on the real-world dataset MIMIC-III. The results show that our model outperforms the baseline approaches.

## Related work

There are mainly two groups of related works: Clinical time series data mining and clinical text mining.

### Clinical time series data mining

EMR data is a collection of patients’ clinical event tables with timestamp. Most existing works focus on mining the relationship and medical statistics by modeling clinical event outcomes.

Because of the effective solution to the problem of long-term dependence, Long Short-Term Memory Neural Network (LSTM) [7] is chosen to tackle with medical series data [8–10], since the PhysioNet Challenge 2012. Harutyunyan et al. [4] provided researchers with the data preprocessing standard on MIMIC III database [11]. According to the acute physiology score table in Apache II, they selected the corresponding characteristics and used LSTM model to deal with four tasks including in-hospital mortality prediction, decompensation prediction, length-of-stay prediction and phenotyping. Choi et al. [12] conducted multilevel medical embedding model, which consists of treatment level, diagnosis level, visit level and patient level, to improve the performance of prediction tasks. They found that the effectiveness of the model can be improved only by using the internal structure of EMR without introducing external knowledge.

As the attention mechanism is proposed [13], more and more people employed attention to capture the dependencies within a neighborhood of the sequence. Song et al. [14] only used masked multi-head self-attention and position encoding in SAnD architecture, which were applied to determine the dependence of different information and maintain the order of the sequence. Ma et al. [15] introduced and modified multi-head self-attention layer into the multi-channel GRU [16] to extract personal healthcare context. Furthermore, they leveraged a cross-head decorrelation to enhance the peculiarity of the different heads. Their called ConCare framework has been proved to have better performance in in-hospital prediction tasks. Although more and more numerical information is taken into account, it is still limited to the examinations performed during the admission period. Information of reference significance such as the past disease history has not been mentioned. This is not enough for the actual treatment process, especially for chronic patients.

### Natural language processing in medical text

Some researchers try to apply Natural Language Processing (NLP) to solve the medical tasks. Grnarova et al. [17] proposed a convolutional document embedding approach and evaluated it on the clinical notes from the MIMIC III. Agrawal et al. [18] extracted the date of the medical events from the clinical text and label the timeline of the documents. Cai et al. [19] employed the attention mechanism to the continuous bag of words (CBOW) [20] model to learn clinical concepts and their temporal scopes. The relations among the medical concepts are extracted and categorized influences of the concepts into three types with the help of doctors: stable influence, peak influence and sequela influence. Gehrmann et al. [21] used Convolutional neural networks (CNN) for medical text classification and compared it with other most used basic models in NLP. They proved the superiority of CNN in the phenotyping tasks and evaluated the interpretability of the proposed method by calculating the most common phrases for predicitons.

Mullenbach et al. [22] presented a convolutional neural network with attention, which is called CAML, to predict medical codes from the clinical notes and showed an interpretability evaluation. The attention weights are applied to calculate the likelihood of each medical code. Following the same idea, Darabi et al. [23] learned the code representation with a Skip-gram model which is based on transformer network and trained a BERT model on the clinical notes leading to the results with time-stamps. The patient embedding obtained demonstrated the effectiveness of the utilization of the unstructured text data. Obviously, the processing of clinical text makes the model enhance the interpretability of the results and the history information contained in the text cannot be replaced by other data in the database.

### Multi-modal learning

Moreover, multi-modal learning has been shown to be beneficial to prediction tasks in many fields such as traffic flow prediction, recommendation systems and also EMR data mining. According to the diversity of influencing factors of traffic flow, researchers [24–26] integrated Point of Interest (POI), weather and time trend characteristics as additional information into the prediction of vehicle trajectory time series data. They confirmed that extra information improved the validity of the prediction results. The network platform can accurately recommend products to users by integrating the users’ operation sequence data with the description information of platform products or short comments of users [27, 28]. Khadanga et al. [6] merged clinical time series embedding and clinical notes embedding together to improve the performance of the model. They are currently the only people who combine time series information with text information and proved its effectiveness. Although both the outcomes of medical examination and clinical notes are taken into account, they did not explore the different dependences of medical texts on different type of patients. In this paper, inspired by them, we made further improvements to the multi-modal learning algorithm proposed by them.

## Methods

**Fig. 2:**
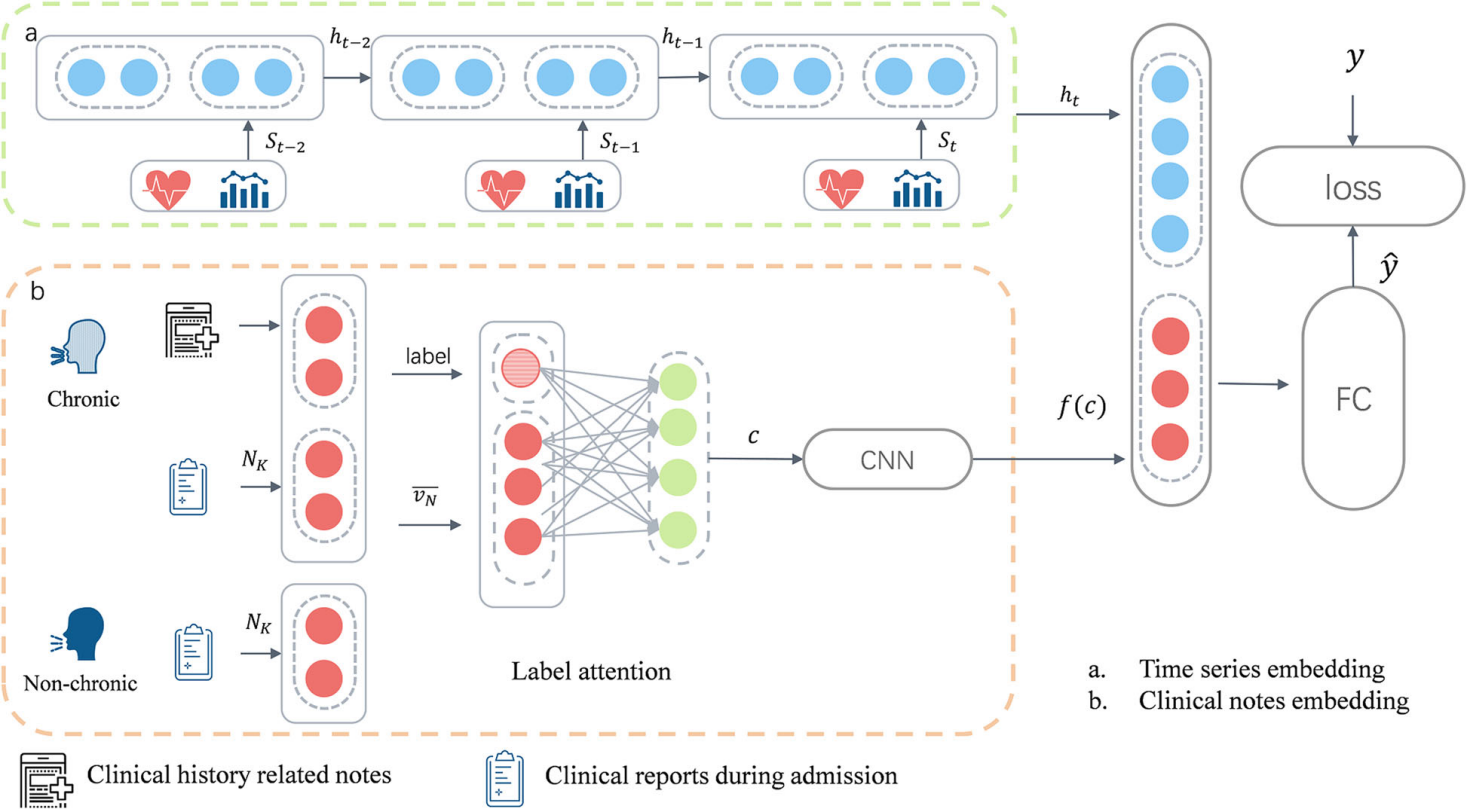
The architecture of the proposed model. **a** is the time series embedding and **b** is the clinical notes embedding. For chronic patient clinical notes include clinical history related notes and clinical reports during admission, while non-chronic patients only consider the later one

In this section, we provide details of our proposed model. As shown in Fig. 2, the approach consists of two major components: Time series embedding model and Clinical time series data mining. We first describe the definition of the mortality task, some notations and overview of the approch. Then, we present the input feature representation of the time series and clinical text. Next, we describe the Time series embedding model and Clinical time series data mining. Finally, we present the loss function of the approach.

### Problem definition

In-hospital mortality prediction task is to predict whether the patient died in 48 hours after the admission of the patient. As depicted in the Fig. 1, the medical event series for every patients is denoted as *S*_*P*_=(<*S*_1,*t*_,⋯,*S*_17,*t*_>∣*t*∈[1,2,⋯,*T*]), in which *S*_*i*,*t*_ represents the *i*-th feature of the *t*-th record and total 17 features according to the acute physiology score table in APACHE II. The features extracted from the dataset for each patient are shown in the Table 1. And $\phantom {\dot {i}\!}N_{P}=\{ N_{with48h}, N_{{history}_{i}} \mid i \in [1,2,\cdots,K] \}$ denotes the clinical notes of patients, where *N*_*w*_*ith*48*h* represents the clinical notes of 48 hours before discharge and *N*_*h*_*istory*_*i*_ represents the ones before 48 hours. And *N*_*h*_*istory*_*i*_ is the *i*-th doctor note out of *K* history related notes. In particular, the clinical notes of chronic patients contain both parts, while non-chronic patients only contain the former part. During the training, a series of {<*S*_*P*_,*N*_*P*_>∣*P*∈(1,0)} are given to train the model, where *P* is defined as the mortality label in 48 hours, which means whether the patient dies in hospital before discharge. In the process of prediction, the death labels are predicted according to the given new <*S*_*P*_,*N*_*P*_>. It is worth noting that because the physical indicators of minors and adults are inconsistent, patients under 16 years old are not be considered in this paper.

**Table 1:** Features from the MIMIC-III for each patient

Feature	MIMIC-III table
Capillary refill rate	CHARTEVENTS
Diastolic blood pressure	CHARTEVENTS
Fraction inspired oxygen	CHARTEVENTS,LABEVENTS
Glascow coma scale eye opening	CHARTEVENTS
Glascow coma scale motor response	CHARTEVENTS
Glascow coma scale total	CHARTEVENTS
Glascow coma scale verbal response	CHARTEVENTS
Glucose	CHARTEVENTS
Heart Rate	CHARTEVENTS
Height	CHARTEVENTS
Mean blood pressure	CHARTEVENTS
Oxygen saturation	LABEVENTS
Respiratory rate	CHARTEVENTS
Systolic blood pressure	CHARTEVENTS
Temperature	LABEVENTS
Weight	CHARTEVENTS
pH	LABEVENTS

The architecture of our proposed model consists of two parts: Time series embedding model and Clinical time series data mining. The temporal events such as lab test results are learned by Time series embedding model and the text in clinical notes are captured by Clinical time series data mining. Both of temporal feature vectors and text vectors are concatenated and fed to a fully connected layer to predict the mortality labels. The details of the model are described below.

### Time series embedding model

Time series embedding model learn the temporal representation of the patients with 17 different concepts mentioned above. In the case of several measurements in the same hour, the average result of the concepts is employed to keep the temporal value frequency of 48 hours before discharge. Same as [6], time series data is modeled by LSTM [7]. LSTM is a neural network structure which can learn the sequential relations and long-term temporal dependencies in the time series data. This function is achieved by the gate and state of the network, in which the forget gate is the key to decide whether to delete the previous state or not. In this paper, the input state at time step *t* is *S*_*t*_ and let *h*_*t*_ represents the current hidden state. And the last hidden state of the LSTM will be the time series embedding for further prediction.

1 $$ h_{t}=LSTM(S_{P},h_{t-1})  $$

### Clinical notes embedding model

As shown in Fig. 2, the clinical notes are extracted to improve the effectiveness of the prediction. Patients are divided into two groups, which are people with chronic diseases and one with non-chronic diseases, to further deal with the clinical history information in the notes. Then the context feature module with label-aware attention is developed to learn the representation of the patients’ clinical notes.

**Clinical Notes Processing for Chronic Patients:** There are several types of clinical notes in the database. In more detail, there are more sections in these notes, which consist of clinical history, history of present illness, past medical history, allergies, family history, social history, service and so on. In previous studies, whether a section of patients’ clinical notes will be extracted is determined by the filling time of the chart. If the chart time is within 48 hours, it will be taken into account. In contrast, the time of filling chart for the clinical history related sections in the NOTESEVENT table are always before 48 hours. So, they are not considered during the extraction of notes although they are important for diagnosis. Therefore, it is particularly important to choose appropriate notes as additional information.

In addition, according to the length of time of onset and duration of disease, diseases can be divided into acute diseases and chronic diseases. There are two scoring sections in APACHE II correspondingly: Acute Physiology and Chronic Health Evaluation. In chronic health evaluation section, patients are given extra points when suffering from chronic diseases such as liver, cardiovascular disease, respiratory disease, kidney disease and immune function suppression. At the same time, Buchan et al. [29] studied on the use of clinical notes to predict coronary artery disease. They found that clinical narratives, which mean the chief complaint and other clinical history related information, are of great significance for the prediction of the disease, and it may be necessary to consider medical records of more than 12 months or even longer periods. According to the review of Sheikhalishahi el al. [30], many researchers have reached similar conclusions in their studies on chronic diseases. This shows that for chronic patients, the medical history information has a great influence on the evaluation. According to the past diagnosis records and clinical records of patients, chronic patients often have persistent complications or be affected by diseases. However, acute patients have a short onset period and have little continuous impact on their bodies, and the possibility of inducing other diseases is very low.

Taking this as a guide, the related sections are extracted from the notes as historical information of chronic patients, some of which are shown in Table 2, while non-chronic patients do not consider these cases. This means that information related to the disease history from the beginning of the medical record to the present is used as the initial information of the clinical notes for chronic patients. For other notes during admission, the relevant text within 48 hours will be extracted according to the table recording time. Therefore, we first identify the chronic patients based on the ICD 9 chronic disease name and code. With this result, the patients in the data set can be effectively divided into chronic patients and non-chronic patients. For non-chronic patients, we only include clinical notes within 48 hours before discharge. For chronic patients, clinical notes since the patient was admitted to hospital will be introduced in addition. All these clinical notes are fed to the label-aware CNN for training.

**Table 2:** Description of some medical history information added in this paper

Section name	Description
Clinical History	Patients’ clinical treatment history.
History of Present Illness	The patients’ basic condition and previous treatment plan.
Past Medical History	Treatment, examination, medicine taken before admission, etc.
Allergies	Patients’ allergic history.
Family History	Family history of chronic diseases.
Chief Complaint	The patients’ self-reported pathogenesis process and symptoms.
Major Surgical or Invasive Procedure	The treatment and invasive surgery that the patients had received.

**Label-aware CNN for Clinical Notes:** To capture the contributions of the different sections from clinical notes and extract text features more effectively, a convolutional neural network with label-aware attention module is introduced to learn the joint embedding of clinical notes and mortality label similar to [31], from which different contributions of the notes to mortality results can be learned. For medical related predictions, people want to know which paragraph of text has a main impact on the final prediction results. The algorithm proposed by them is to learn the degree of similarity between different parts of the text and the final prediction labels, that is, the degree of association. Therefore, we introduced this module to learn the contributions of different parts of the clinical notes on the prediction of patient mortality.

The clinical notes *N*_*P*_ are embedded to a text vector $\overline {v_{N}}$ by projecting every word with Word2Vec [20], which is trained in the PubMed – a professional medical citation datasets with amount of science journals and books. Then given the clinical notes feature representation of a patient $\overline {v_{N}}\left (\overline {v_{N}}\in \mathbb {R}^{D}\right)$, in which *D* is the size of the vocabulary, the projection for a note feature in the joint space can be derived as $v_{N}=W^{v1}\overline {v_{N}}\left (v\in \mathbb {R}^{P}\right)$. In the same way, the projection of the label embedding $\overline {v_{m}}\left (\overline {v_{m}}\in \mathbb {R}^{O}\right)$ to joint space $v_{m}=W^{v2}\overline {v_{m}}\left (v\in \mathbb {R}^{P}\right)$, where *O* is the number of the label class. The label for the mortality prediction task is denoted as a one-hot embedding. For more details, $W^{v1}\in \mathbb {R}^{P\times D}$ is the transformation matrix that maps the note vectors into the joint embedding and *P* is the dimensionality of the joint space. In the same way, $W^{v2}\in \mathbb {R}^{P\times O}$ projects the label vector into the joint embedding space. The compatibility of the clinical note vector and label vector are obtained by *G*=*v*_*N*_⊗*v*_*m*_. For more detail, for the *i*-th word in the sentence, *G*_*i*_ represents the compatibility between the word and labels. The similarity between the word and the label is:

2 $$ u_{i}=ReLU({G_{i}}{W_{i}}+b_{i})  $$

where *W*_*i*_ and ${b_{i}}\left (b_{i}\in \mathbb {R}^{O}\right)$ are learned parameters. And the total max-pooling output of all *u*_*i*_ is *m*.

In the joint embedding space, it is expected that the dependency between the word in notes and mortality labels to be more reflective of semantic closeness between clinical notes and mortality results. The effective way to measure the dependency is to add an attention layer. According to the definition of the attention [13]:

3 $$ Attention(Q,K,V)=softmax\left(\frac{{Q}{K^{T}}}{\sqrt{d}}\right)V  $$

where the query *Q*, key *K*, and value *V* are set to the joint vector embeddings *G*, and *d* is the embedding dimension. So, the label attention weights for *i*-th words are denoted as:

4 $$ {\alpha}_{i} =\frac{exp({m_{i}})} {\begin{aligned} \sum_{j=1}^{L} exp(m_{j}) \end{aligned}}  $$

The total attention score *α*=*Attention*(*m*). The joint embedding is finally defined by multiply the word embeddings and the attention score:

5 $$ q={\alpha}v_{N}  $$

The label attention here describes the relationship between the words and mortality label and considers which words have been attended to in the clinical notes extracting process. Then the output of the embedding layer is fed to a convolutional layer to further prediction. The *i*-th text feature extracted by the convolutional is obtained by:

6 $$ c_{i}=f({W_{q}}q_{i} + b)  $$

where *W*_*q*_ and *b* are weight matrix and bias respectively. And *f* is an activation function, which is usually nonlinear. The output of the CNN layer will be concatenated with the temporal embedding to further predict.

### Prediction component

To predict whether a patient will die after 48-hour admission, the time series embedding and clinical notes embedding are joined together by concatenating them. So, the concatenate vector is denoted as:

7 $$ z={h_{t}}\oplus c  $$

where *c* is the output of a CNN layer. The concatenate embedding *z* is finally fed to a fully connected network to predict a $\hat {y}$ for every patient. The prediction function is:

8 $$ \hat{y}=\sigma ({W_{f}}z + b_{f})  $$

where *W*_*f*_ and *b*_*f*_ are learnable parameters. And *σ* is a sigmoid function, which is defined as *σ*(*x*)=1/(1+*e*^−*x*^).

The goal of the joint embedding is to minimize the similarity between the clinical text representation and patients’ labels. So, the cross entropy (CE) loss and joint embedding objective are combined to train the model. The Tensorflow [32] and Keras[33] are used to implement the proposed model, in which the CE loss is defined as:

9 $$ CE({y_{i}},{\hat{y}_{i}})=-{{\Sigma}_{i}}{y_{i}}{log({\hat{y}_{i}})}  $$

where $\hat {y}_{i}$ is the prediction labels and *y*_*i*_ are the ground truth labels. Note that the labels are not used in the test set, because we already get the similarity scores between the words and labels. The loss function used in training process is:

10 $$ loss(\theta)=CE({y},{\hat{y}})+{\frac{1}{k}}{\begin{aligned} \sum_{n=1}^{l} CE\left({y_{k}},\sigma \left(c_{k}\right)\right) \end{aligned}}  $$

where *θ* represents the all learnable parameters in the model and *σ*(*x*) is also the sigmoid function in the clinical notes embedding model. And this regularization means the penalty of the joint embedding, which results in the interpretability. Furthermore, the optimization in this implement is Adam [34].

**Quantifying Section Importance:** The label aware CNN mentioned above describes the contribution of each word to the final prediction, and as mentioned before, usually the clinical notes of the patient contain many sections, such as nursing notes, clinical history, family history and so on. So, it is necessary to capture how the contribution of each section can be more effective to know the role of each section for prediction. To further measure the contribution of different sections to the mortality prediction, we define the importance score as:

11 $$ Score \left(N_{i,i+l}\right)=\frac{{\alpha}\left(N_{i,i+l}\right)}{\begin{aligned} \sum_{n=1}^{K} \alpha\left({N_{n}}\right) \end{aligned}}  $$

where *α*(*N*_*i*_) is the attention score of *i*-th word. And for each section of the clinical notes, suppose the. For every patients notes, suppose the total number of words is *n*. The first character of each section is the *l*-th, and the total number of sections is *m*. The attention score here are the obtained by training and each of them can be interpreted as the contribution to a specific mortality label. Therefore, the importance of each section can be derived from the sum of the importance of all its words in the entire notes. The effectiveness of this equation as scoring function is visualized and verified in the Results section.

## Results

### Dataset

In this paper, the MIMIC-III [11] dataset is used to evaluate the proposed approach. There are about 50,000 patients information in the ICU from Beth Israel Deaconess Medical Center between 2001 and 2012, where the sensitive data of people has been encrypted. The age distribution of patients is very wide, and only patients over 16 years old are selected because of the special nature of the minor’s physical standards. The database contains the patient’s demographic information, the treatment received during admission, the results of the physical examinations performed, clinical notes and so on. The LABEVENTS table, CHARTEVENTS table and NOTESEVENT table are the main data source for this paper. All these data are merged by *stays*_*i*_*d* which means the patients’ hospital stay id. These features include the temporal features and events features.

**Fig. 3:**
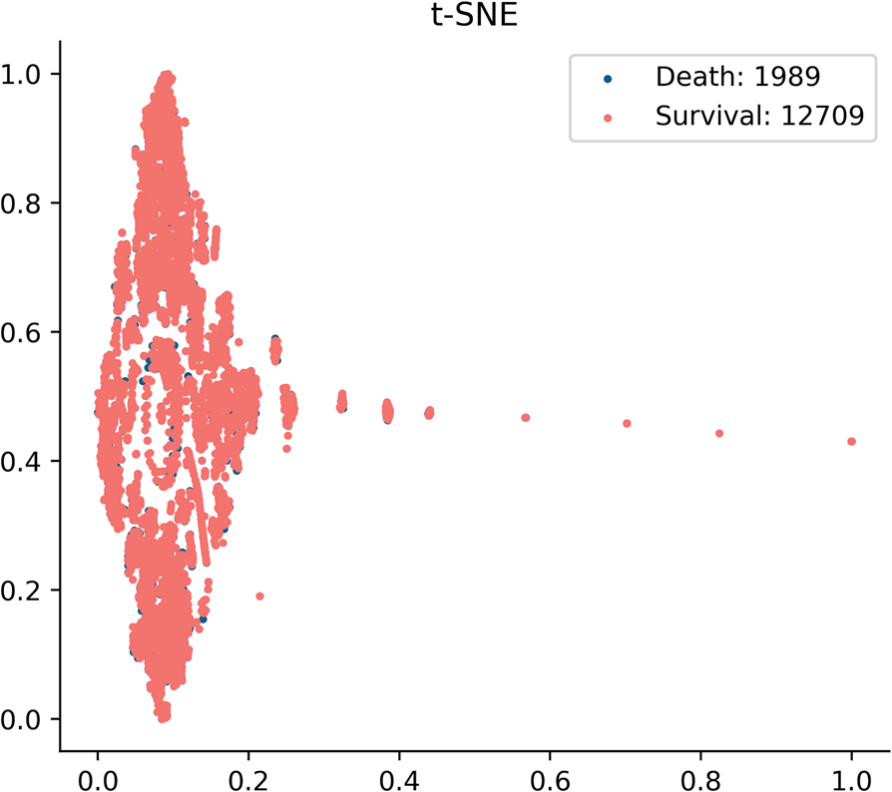
The t-SNE visualization of the collected data. The red points represent the survival patients and the blue ones are death patients

In the experiment, the data for mortality prediction is collected following the benchmark research [4]. Then the patients stayed in the ICU for at least 48 hours are selected same as the research [6]. The distribution of the collected data for prediction is shown in Fig. 3 which is visualized by t-SNE [35]. It can be seen from the figure that the data used in the experiment has a problem of label imbalance. Only a small number of people eventually died within 48 hours, and most people survived.

### Dataset analysis

**Fig. 4:**
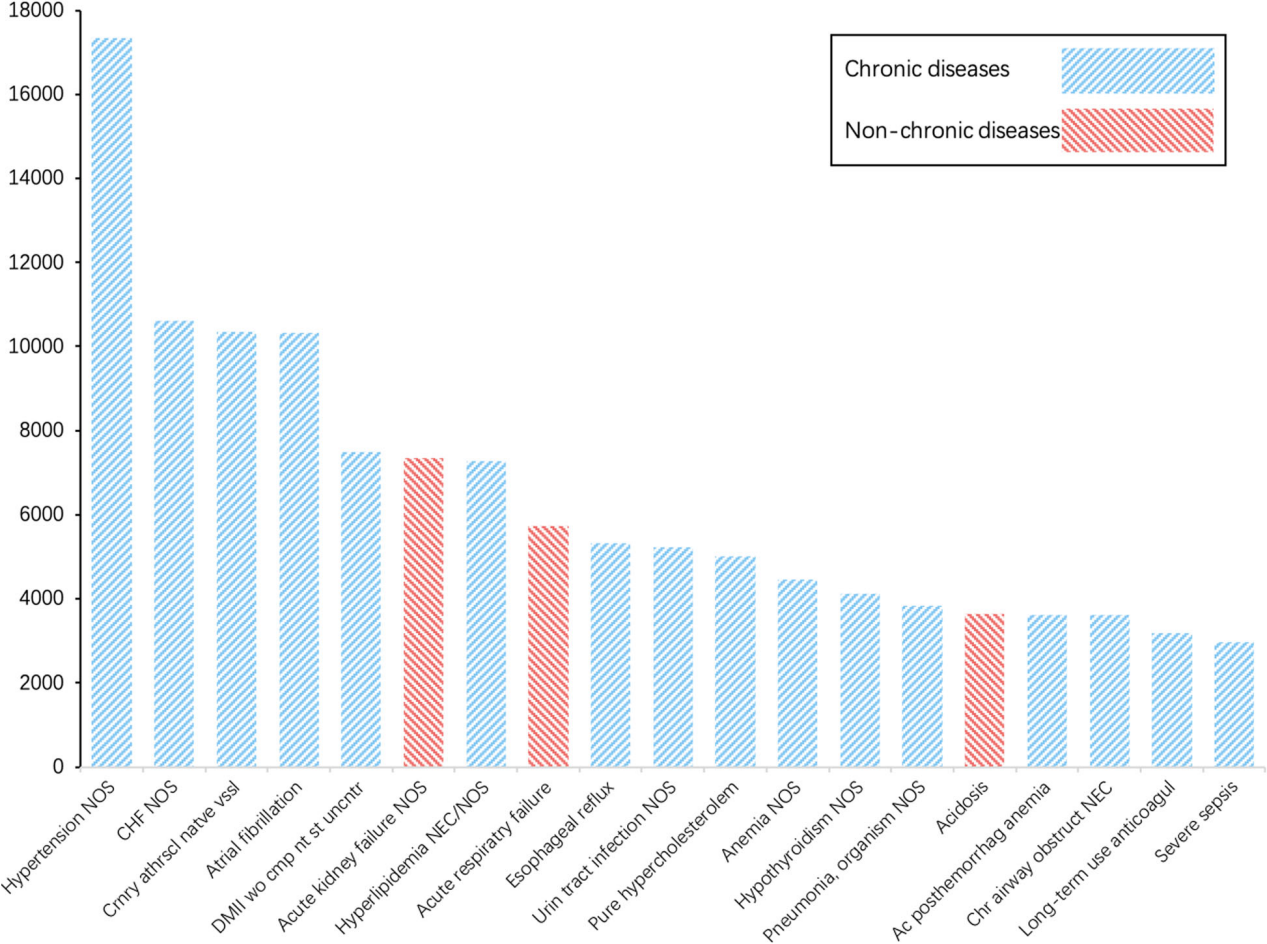
The distribution of twenty diseases with the largest number of patients

In the MIMIC-III dataset, patients suffer from various diseases. According to the diagnosis results of patients, we make a statistical analysis of the top 20 diseases with the largest number of patients in the diagnosis results. As shown in the Fig. 4, patients with hypertension are the most in the data set. This is because most patients in ICU have basic diseases and the first five items are the most common chronic diseases in reality. On the whole, only three diseases are non-chronic diseases. And the number of acute patients is very small compared with that of chronic patients. It means that the majority of patients suffer from the chronic diseases. At the same time, it shows that a large number of clinical historical data of chronic patientshave not been taken into account in the previous research. Therefore, the introduction and processing of this part of information are considered in this work.

### Evaluation metric

As mentioned in above section, the dataset used is very imbalanced. According to the research [36], it may not be appropriate to use precision or recall alone that is often used as evaluation indicators in the classification. The Precision-Recall is more informative for classification on the imbalance datasets. So, Area Under Precision-Recall (AUCPR) and Area Under Receiver Operating Characteristic (AUCROC) are chosen to evaluate the proposed method.

AUCPR is defined as the area under the curve, where recall and precision are the abscissa and ordinate, respectively. And AUCROC is also the area under the curve, but abscissa is False Positive Rate (FPR) and ordinate is True Positive Rate (TPR) instead.

### Methods for comparison

Our model is compared with the following methods, and the performance of them will be discussed later. **LSTM** [4]: The time series data selected in this paper is fed to the pure LSTM model to predict the probability of death in 48 hours after admission.**Clinical Notes Only (CN)**: This paper [22] used CNN to predict medical codes of the patients. Similar to the cited paper, the clinical text representation is fed to a CNN model to predict the mortality.**Multimodal with Clinical Notes (Muti-CN)** [6]: This method combine the LSTM time series embedding and clinical notes representation to address the task. CNN is used to extract features from the clinical notes. Note that the notes here are same as our preprocessing.With these three methods, we can study the effect of different part of our proposed multimodal approach. We use the same time series embedding for the LSTM, Multi-CN and our method. We also studied the influence of the processing of the clinical notes of chronic patients we proposed:**Multimodal with Label aware CNN (Multi-atten):** In this variant, we treat chronic patients and non-chronic patients in the same way. Both of the clinical reports during admission and clinical history related text are considered and fed to the label aware attention CNN model to train.**Multimodal with Label Attention for chronic patients (Multi-atten-chronic):** our proposed model, which consists of processing of the chronic patients history information and label aware CNN model.

### Setup and performance comparison

**Setup:**All these experiments were run on a NVIDIA RTX 2080Ti GPU. The timestep is set as 1.0 in LSTM. The output dimension of LSTM is set to 256. The max length of the clinical notes is 1000 and if the length of the text is less than it zero padding will be used. Batch normalization is used, and the batch size is set to 5. The learning rate is 0.0001. The early-stop round is set to 20 and the max epoch is 100.

**Comparison with the methods:**Table 3 shows the performance of our proposed approach and the methods mentioned above Multi-atten-chronic achieves the highest AUCPR and Multi-atten achieves the highest AUCROC. Comparison results can be divided into three parts. LSTM is a baseline that only considers time series data and only the clinical notes are considered in CN. Multi-CN is by far the best model considering both of them at the same time. More specifically, we can see that the LSTM and the clinical notes only methods are the lowest and similar in the comparison in both of AUCPR and AUCROC. This is because the clinical notes contain a part of physical examination result data, which will be duplicated with the time series data. The result of multi-cn is better than that of considering only one kind of data, which indicates that the fusion of time series data and clinical notes is effective. And our model has been improved on this basis. The performance of Multi-atten proves that the overall performance of our model has been improved after joining label-CNN. Furthermore, the clinical notes of chronic patients are treated differently, and the overall effect of the model has been improved. This comparison proves the effectiveness of our proposed model.

**Table 3:** Comparison with baselines

Method	AUCPR	AUCROC
LSTM [4]	0.487	0.844
CN	0.432	0.835
Multi-CN [6]	0.559	0.854
Multi-atten	0.556	0.861
Multi-atten-chronic	0.562	0.857

### Visualizing contributions of the clinical notes

**Fig. 5:**
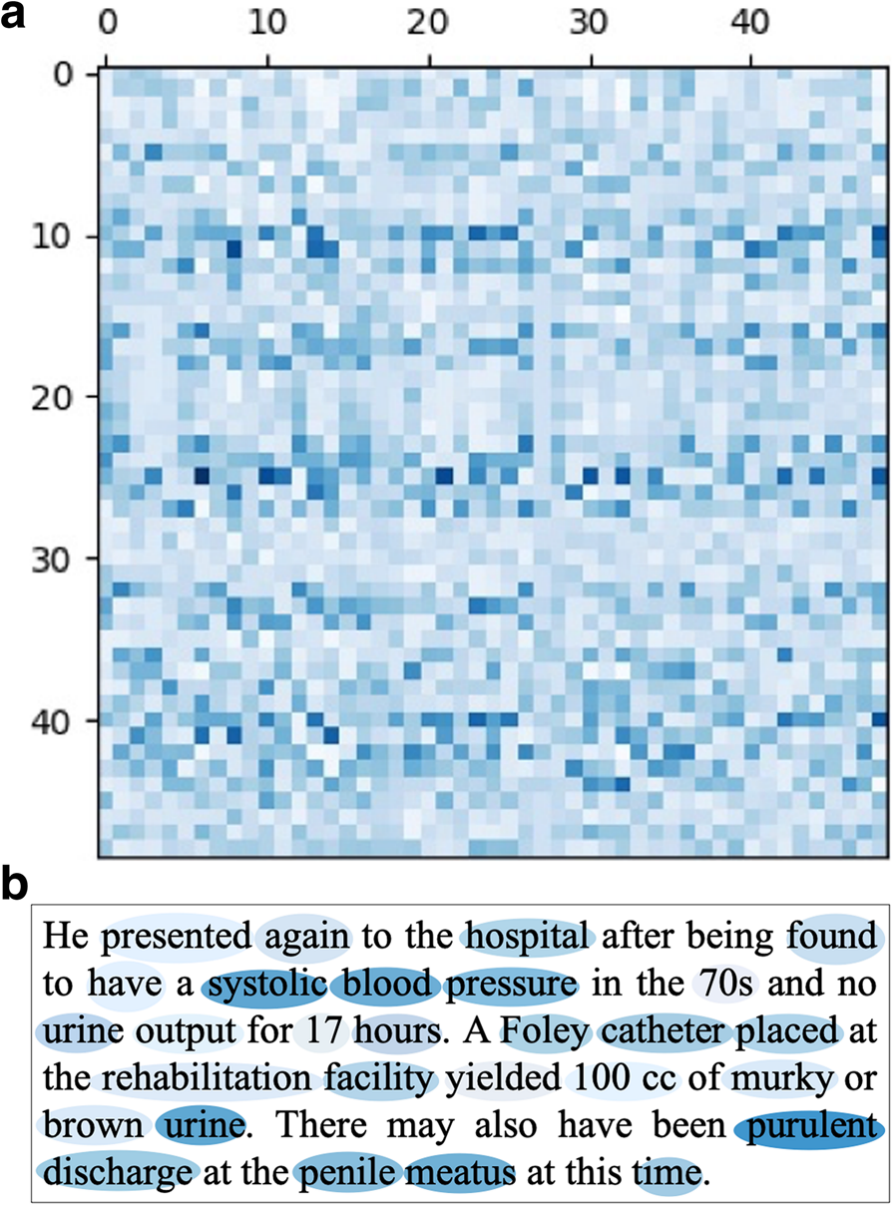
Text heatmap and visualization sample. **a** is text heatmap of the learned clinical notes. The darker the blue is, the more important the words to the context. And **b** shows that the color of color block is related to the attention score

Figure 5 shows the visualization of the dependencies of the clinical notes text to the death and survival prediction. The darker the color of the words, the greater the contribution to the prediction result. That means the words with white colors are less important in the sentence, while the dark blue words denote the evidence for death of the patients. Here the rows represent the sentences in the clinical notes while the columns are the joint embeddings of the words. The first fifty words in clinical notes with clinical history related notes of a chronic patient are presented in the Fig. 5a. According to the meaning of the rows and the columns, it can be seen that the color blocks of the words show the degree of contribution and dependence of each word to the labels.

We present the attention scores with the actual text in Fig. 5b. Also, the shade of the blue indicates that how much the words contribute the final label. The darker words are more important in the death label prediction. It should be noted that the importance score for stop words is not calculated. In this case, the color of “systolic blood pressure” is almost the deepest. Similarly, medical related nouns such as “foley catheter”, “urine” and “purulent discharge” are also darker in color. These are the parts that doctors compare in actual diagnosis, which shows that our model has paid attention to these places effectively. At the same time, words like “rehabilitation facility” are also important for prediction.

**Fig. 6:**
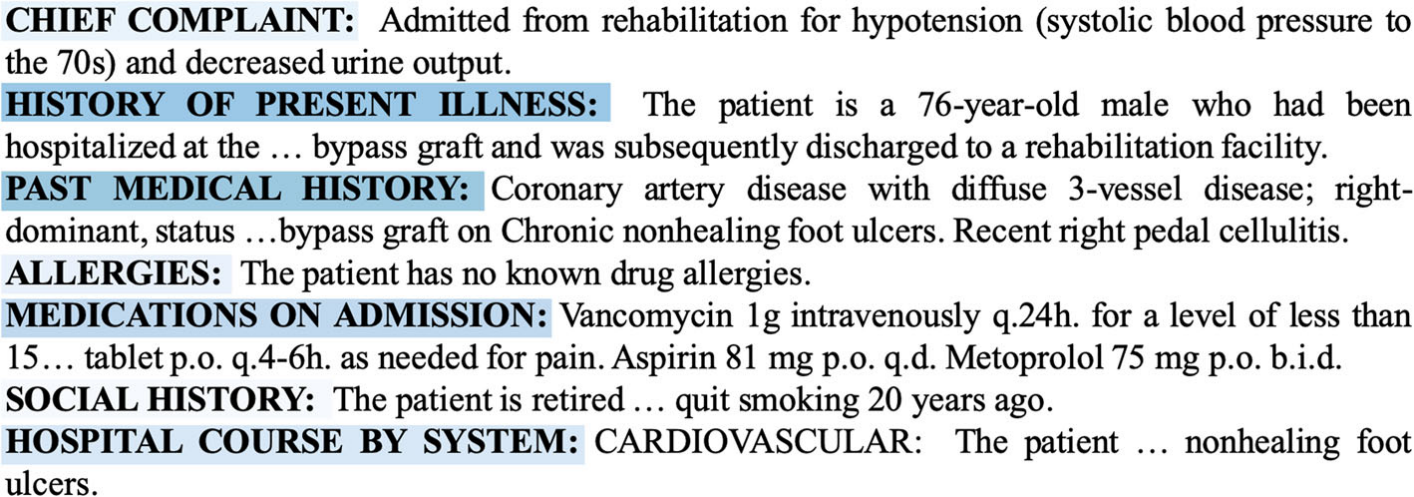
The importance sample of the clinical note sections. The darker the blue is, the more important the section to the prediction

The importance scores of different sections are showed with the actual text in Fig. 6. The representation here is the same as Fig. 5, which means the darker sections are more important. In this case, the color of “HISTORY OF PRESENT ILLNESS” is almost the deepest and the color of “SOCIAL HISTORY” is the lightest. The characteristic of section with deep color is that it contains more medical related contents. Correspondingly, it contains more related words, which leads to a higher score. The visualization results of these two figures fully show the effectiveness of scoring function.

### Hyper-parameter analysis

In this section, the influence of hyper-parameters is analyzed. During the training process, the early stopping method is adopted, and almost all models showed no significant performance changes after the 50th epoch. Therefore, for different hyperparameters, we compare the model performance with every 10 epochs during the training process.

**Fig. 7:**
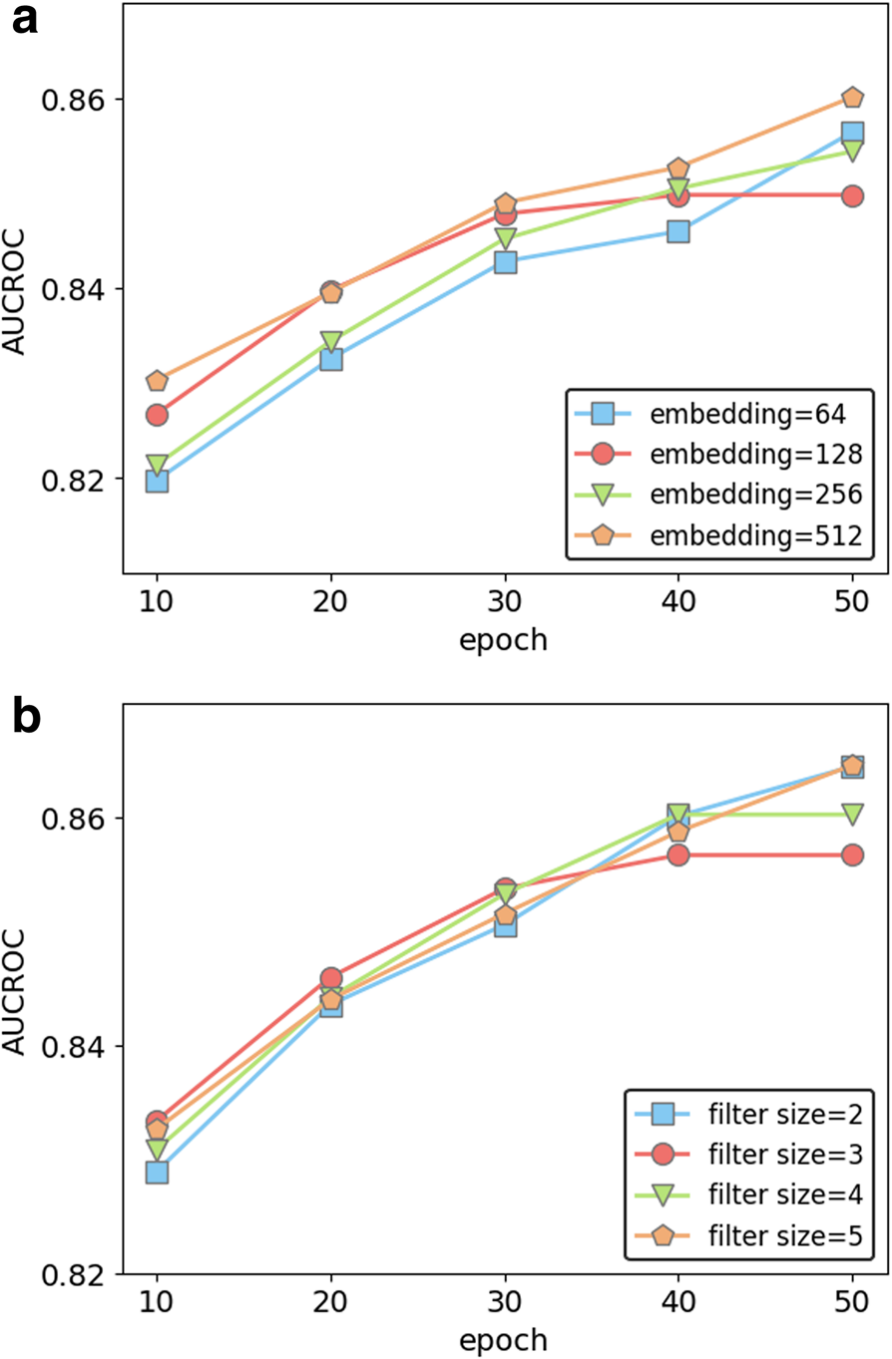
AUCROC of different CNN hyper parameters every 10 epochs

**Fig. 8:**
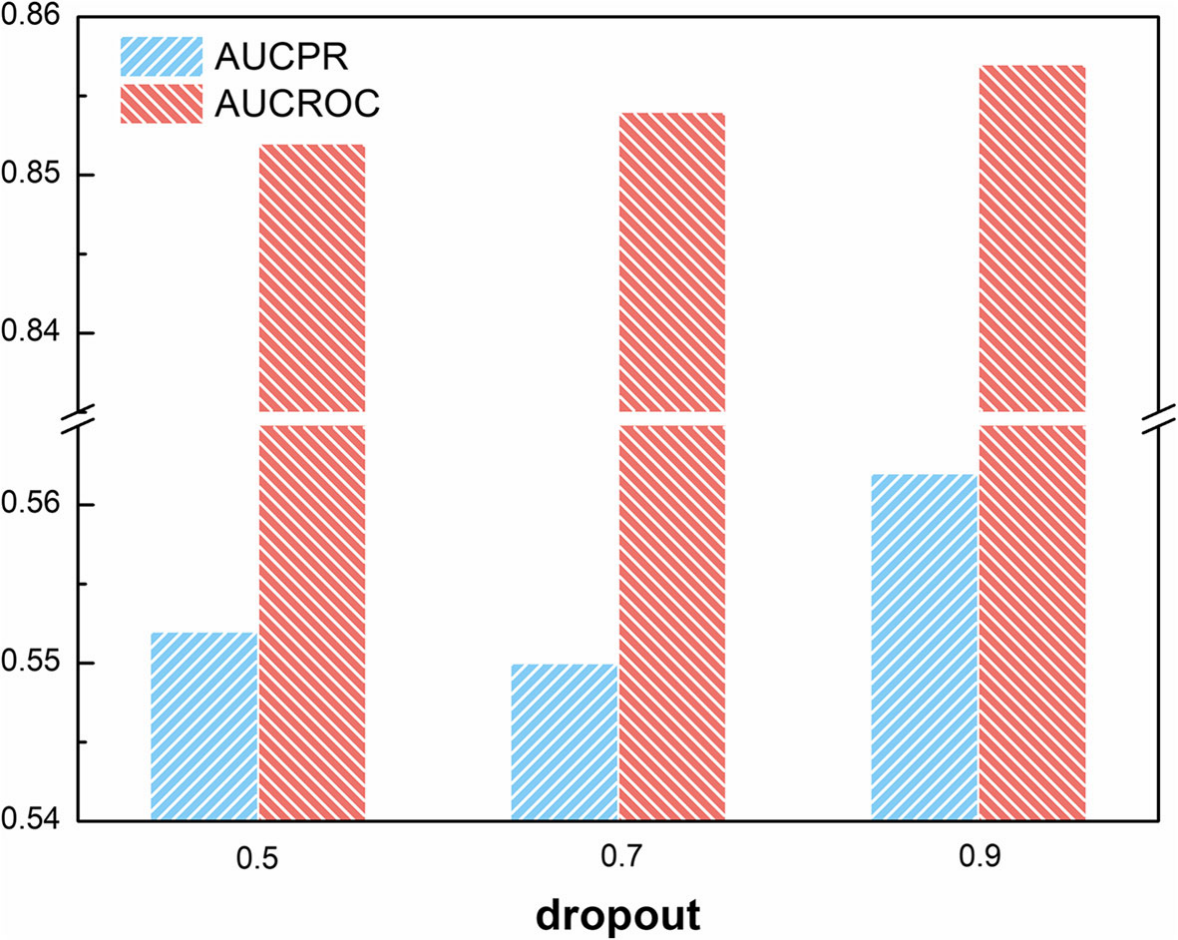
The influence of dropout

The influence of hyper-parameters of CNN is shown in Figs. 7 and 8. The impact of input embedding dimensions in CNN layer is depicted in Fig. 7a, from which we can see that AUCROC has improved with the increase of embedding dimension. Because the input embedding represents the dimension of the word vector, the higher the dimension, the better the representation of the word. In the end, the improvement of the notes feature brought an improvement in the prediction results. Another important hyper-parameter in CNN is the filter size. Unlike images that use pixels as a unit, word vectors require a certain dimension to extract a complete word or phrase representation. Figure 7b describes the impact of filter sizes commonly used in text classification CNN model. It can be seen from the figure that when the filter size is 2 or 5, the model performs almost the same and the effect is relatively best. Finally, we observed the experimental results of different dropout. Finally, we observed the experimental results of different dropout. It can be seen from Fig. 8 that when dropout is 0.9, the model reaches the best at both values. Therefore, we set dropout to 0.9 during training.

## Discussion

We first analyzed the distribution of non-chronic and chronic patients in the dataset. It can be seen from the results that chronic patients account for the majority in the MIMIC-III data set. Therefore, it is necessary to treat the data of chronic patients differently in the process of predicting mortality. The comparison results in Table 3 show the advantages of the multimodal learning methods. However, multi-CN does not consider the different contributions of the clinical notes and lacks clinical history related notes. In the Multi-atten model, both of chronic patients and non-chronic patients are treated with clinical notes which include the clinical history related text. The result of the Multi-atten demonstrates that the label aware attention layer makes the model more sensitive to the context of the clinical notes compared to the multi-CN. Furthermore, the additional introduction of clinical notes to chronic patients can respond to the different needs for clinical history information. The process of this operation is closer to the diagnosis process of actual doctors. So, the AUCPR as a measure of the overall performance of the model reached the highest. This proves that it is necessary to treat the clinical notes of chronic patients and non-chronic patients differently. At the same time, for chronic patients, it is necessary to add historical information.

In the visualization experiments, it can be seen in the Fig. 6 that the total contributions of the history of present illness and past medical history sections are the highest in these seven sections. Meanwhile allergies and social history are the least important ones. The reason is that there are few words in this part. And most of them do not contain professional medical terminology related vocabulary. Through the visualization of words in Fig. 5, it can be found that medical related words usually have higher attention score. The visualization of the results shows the interpretability of the model with label attention layer and the attention score can detect the words associated with death label. According to this experiment, we can see which part of clinical notes has a practical influence on the death of patients. This not only makes the prediction result more explicit, but also proves the effectiveness of scoring function. This result can tell the main inducement that affects the development of the patient’s illness, which has certain significance for the follow-up study.

To sum up, in the study of electronic medical records, tasks like mortality prediction are the focus of researchers. In the existing research, to our best konwledge,few people use time series data features and text features at the same time. With the rapid development of multimodal learning models, we consider making full use of these two types of data. In this article, we further extend the model on the basis of existing research [6] and hope to improve its performance.

For the most important interpretability aspect in medical research, we propose a simple but very effective scoring function. We can find which section in the clinical notes has the most influence on the final prediction results through this function. These sections have very specific medical significance for the patient’s treatment process. The visualization of the influence of sction can provide some auxiliary opinions for the doctor’s diagnosis and treatment process.

## Conclusions

In this paper, a multimodal network are proposed for the mortality prediction by using time series data and clinical text at the same time. The time series data is modeled by LSTM and the clinical notes are learned by a convolutional neural network with label aware attention layer. Furthermore, the history information of the chronic patients is treated with independently and the processing of the reports during admission is the same to chronic patients and non-chronic patients by processing of chronic clinical notes. The model is evaluated on the collected data from MIMIC-III dataset. The experiment results show that our proposed approach outperforms the competing methods. The results are not only better than the existing multimodal model with the best results, but also far more than the model that only considers single type of data. And the visualization results show that the model pays more attention to the vocabulary related to medical process and indicate the interpretability improvement of our model.

Although the APACHE II scoring system is used as feature selection guide, it is still far from the actual diagnosis process of doctors. For future work, it is crucial to introduce the existing medical knowledge to the deep learning model. Knowledge graph is a popular tool to capture the background knowledge, which can be utilized to bring further improvements in several aspects in the model. So, incorporating more medical information is the direction of our future work.

## Data Availability

The datasets analyzed during the current study are available from the Medical Information Mart for Intensive Care (MIMIC-III). More information about MIMIC-III can be found on their website (https://mimic.mit.edu/about/mimic/).

## Abbreviations

LSTM: Long Short Term Memory
CNN: Convoluntional Neural Networks
APACHE: Acute Physiology and Chronic Health Evaluation
EMR: Electronic Medical Records
ICU: Intensive Care Unit
CE: Cross Entropy
AUCPR: Area Under Precision-Recall
AUCROC: Area Under Receiver Operating Characteristic
MIMIC: Medical Information Mart for Intensive Care
